# Emergence Biases in Molecular Evolution

**DOI:** 10.1093/gbe/evag195

**Published:** 2026-07-30

**Authors:** Timothy Fuqua, Nikolaos Vakirlis

**Affiliations:** Institute of Ecology and Evolution, University of Bern, Bern, Switzerland; Swiss Institute of Bioinformatics, Quartier Sorge-Batiment Genopode, Lausanne, Switzerland; Hellenic Pasteur Institute, Athens, Greece

**Keywords:** emergence, bias, evolution, molecular biology, gene regulation, proteins

## Abstract

Biases in molecular evolution can significantly influence evolutionary trajectories. They have been described in a variety of contexts, such as development and mutation, but not for the emergence of new and observable molecular activities. Here, we formalize the term “emergence bias” as the molecular predisposition that, upon mutation, biases a genetic sequence toward or against manifesting new or latent functions or phenotypes. These biases have been observed in previous studies for the emergence of promoters, enhancers, and de novo proteins, but have never been formally characterized as such. In this Perspective piece, we describe these studies and synthesize their findings through the prism of a unifying term, emergence bias, to provide support for this new concept and speculate on its molecular underpinnings. We believe that emergence biases may play an important role in evolution by canalizing which functions can and cannot emerge.

Significance The extent to which new biological functions emerge or become observable as a consequence of mutations can substantially differ between DNA sequences. This work describes examples of such biases in molecular evolution, particularly for the emergence of new regulatory DNA and proteins and speculates on the mechanisms underlying the biases. The authors propose that this phenomenon be described as “emergence biases” in molecular evolution.

## Introduction

There are a number of characterized biases underlying molecular evolution. These include developmental biases where mutations produce an unequal distribution of forms (Arthur 2004), mutational biases where the rates of different types of mutations differ (Cano et al. 2023), and codon biases where synonymous codons are not uniformly encoded (Parvathy et al. 2022). Each of these biases can significantly influence what phenotypic variation is possible, effectively canalizing some evolutionary trajectories and constraining others (eg developmental (Alberch 1985; Alberch and Gale 1985; Braendle et al. 2010; Fuqua et al. 2020; Rohner and Berger 2023), mutational (Stoltzfus and McCandlish, 2017; Storz et al. 2019; Cano and Payne 2020), and codon (Fragata et al. 2018)). Historically, this phenomenon has also been referred to as “the possible versus the actual” (Jacob 1982; Schuster et al. 1994; Stadler et al. 2001).

To describe a bias, one must specify what it is relative to. For example, developmental biases are relative to a default assumption that all forms evolve equally within an observed fraction of a morphospace (Uller et al. 2018). Similarly, codon biases are relative to a null assumption that all synonymous variants are encoded equally (Parvathy et al. 2022). For many cases, however, there is not a clear null or default assumption. When this occurs, biases of one molecule can also be relativized to another molecule. For instance, one could compare the evolutionary potential of a DNA sequence to that of a randomly synthesized DNA sequence (Yona et al. 2018; Lagator et al. 2022; Camellato et al. 2024), foreign DNA (Wong et al. 2020; Luthra et al. 2024), or the reverse version of the sequence of interest (Camellato et al. 2024). The caveat with such relative comparisons, however, is that they cannot preserve all aspects of a tested sequence. For instance, scrambling a DNA sequence may maintain AT content, but does not maintain dinucleotide composition. Arguably, one of the challenges of characterizing biases in molecular evolution is finding the appropriate null models and assumptions.

Furthermore, the directionality of a bias is described using terms such as “drive” (Arthur 2001) or “constraint” (Smith et al. 1985). One potential issue with these terminologies is that “drives” connotes positive selection, while “constraints” connotes negative selection. The terms additionally become problematic when considering pleiotropic traits, where it is not clear which trait is being selected for, and which is a byproduct of selection (Gould and Lewontin 1979). The terms’ distinction, however, is arguably redundant, as a drive toward one phenotype is simultaneously a constraint against another (Arthur 2001). In this Perspective piece, we simply refer to both drives and constraints as “biases” and specify their directionality as either “toward” or “against” a particular trait. Under this view, directionality of a bias is irrelevant to selection.

Finally, throughout this piece, we use the loaded term “function” (Keeling et al. 2019). Here, we adopt a biophysical perspective, referring to function as the ability of a DNA sequence to produce a molecular activity that favors a phenotype under particular conditions. Furthermore, these molecular activities are detectable, and above the threshold of the weak, background activity of the genome (Boyle et al. 2017), and are not necessarily products of selection. By extension, nonfunctional sequences may exhibit weak or “moonlighting” molecular activity (Jeffery 2018), resemble “proto-genes” (Carvunis et al. 2012), or even exhibit the molecular activity under different conditions. Therefore, the apparent binary process of a nonfunctional DNA sequence acquiring a new function through mutations (ie emergence) can also be continuous. For example, a sequence that favors a molecular activity below the detection or classification thresholds can gradually accumulate mutations, but may appear to be a binary process based on our definitions and technologies.

**Fig. 1.**
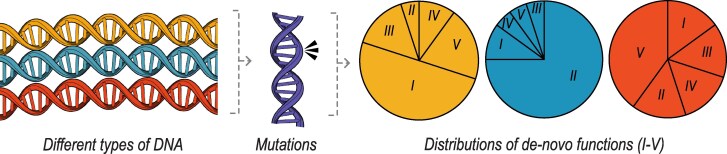
Emergence biases. Emergence bias is a molecular predisposition that, upon mutation, biases a genetic sequence toward or against manifesting new or latent functions or phenotypes. Left: different genetic sequences colored in yellow (top), blue (middle), and red (bottom). Middle: sequences are exposed to mutations. Right: the frequency of new functions, labeled I–V acquired in the mutants. Cartoons are for illustrative purposes only. The majority of mutations will likely not produce any detectable function.

Recent developments in computational and experimental approaches have begun to reveal that the emergence of novel functions is an important aspect of evolutionary innovation (Bornberg-Bauer and Eicholt 2026). Examples of emergence have been described from regulatory DNA (Eichenlaub and Ettwiller 2011; Li et al. 2023) to the emergence of entire protein-coding sequences (Zhao et al. 2024). To the best of our knowledge, there is no formally described term that can apply to the biases underlying such emergence. The term “preadaptation,” as employed by Masel (2006) (most relevant to our work), refers to a variation which, through the action of selection, has been made more likely to result in a new function upon a shift in the environment, eg by the removal of deleterious variants. The term “exaptation” (Gould and Vrba 1982), which covers cases of traits originally evolved for one specific function that are co-opted for a new one, is also linked to the action of selection and strongly associated with organismal evolution. Thus, none of these terms adequately encompasses all biases affecting the emergence of function irrespective of selection. This Perspective piece aims to unite similar ideas and concepts throughout the literature under a missing umbrella term: “emergence bias,” which we define as a molecular predisposition that, upon mutation, biases a genetic sequence toward or against manifesting new or latent functions or phenotypes (Fig. 1). Here, we present evidence for emergence biases across different subfields of molecular biology and discuss the possible mechanisms underlying them.

### Evidence of Emergence Biases

#### Prokaryotic Promoter Emergence Biases

Prokaryotic promoters are DNA sequences that RNA polymerase binds to for initiating transcription (van Hijum et al. 2009). Mutations to nonpromoter sequences can readily create new promoters from as little as single-point mutations (Yona et al. 2018; Lagator et al. 2022; Fuqua et al. 2024; Fuqua and Wagner 2025, 2026). Furthermore, upon synthesizing randomly generated DNA sequences, ∼10% will have promoter activity, depending on their sequence lengths and AT contents (Wolf et al. 2015; Yona et al. 2018; Warman et al. 2020; Lagator et al. 2022).

**Fig. 2.**
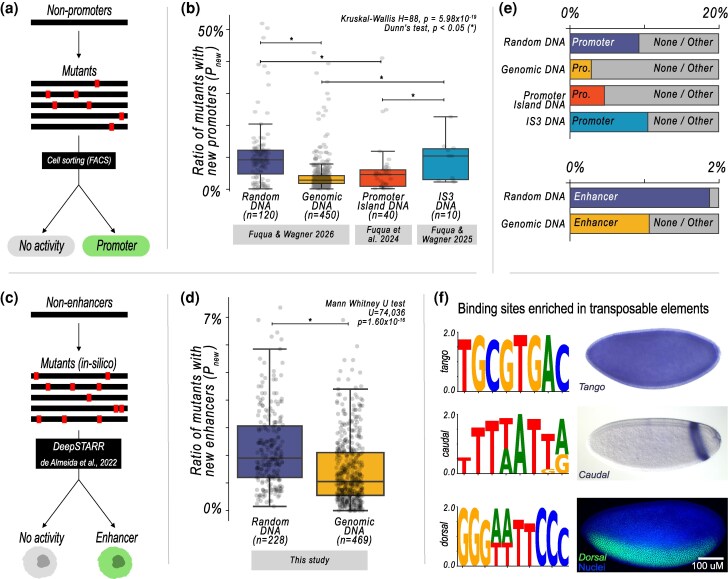
Emergence biases of cis-regulatory DNA. a) Previous works experimentally mutagenize different categories of nonpromoter DNA sequences and test the mutants for promoter activity in *E. coli.* b) The ratio of mutants with new promoters (*P*_new_) for each mutagenized sequence (individual points). Each box plot corresponds to a category of nonpromoter DNA from the studies listed below. Data were all acquired using the same cell sorting and gating strategy (Sort-seq). We test the null hypothesis that the *P*_new_ distributions are the same using a Kruskal–Wallis test (H = 88, *P* = 5.98 × 10^−19^) and use Dunn's post hoc test to identify significant differences between the categories. Horizontal lines with asterisks (*) above indicate *P* < 0.05. c) We randomly mutagenized DNA sequences not predicted to have enhancer activity in *Drosophila* S2 cells and test the mutants for new enhancer activity in silico using the DeepSTARR sequence-to-function model (de Almeida et al. 2022). d) The ratio of mutants predicted to be enhancers (*P*_new_) for each mutagenized sequence (individual points) using DeepSTARR. Each box plot corresponds to a category of nonpromoter DNA (left, randomly generated DNA; right, fragments of the *D. melanogaster* genome). We test the null hypothesis that the distributions are the same using a Mann–Whitney *U* test (U = 74,036, *P* = 1.60 × 10^−16^). See Methods for simulation details. e) We plot the median *P*_new_ value for each category. Note that the *y* axes differ for promoters (top, 0% to 20%) versus enhancers (bottom, 0% to 2%). f) Left: sequence logos for three TF binding motifs enriched in *Drosophila* TEs identified in Villanueva-Cañas et al. (2019). Logos are derived from the Fly Factor Survey database (Zhu et al. 2011) and drawn using LogoMaker (Tareen and Kinney 2020). Right: representative gene expression patterns for *D. melanogaster* embryos at approximately developmental stage 5. Images for Tango and Caudal are in situ hybridizations for the respective transcripts from the Berkeley Drosophila Genome Project (Tomancak et al. 2002, 2007; Hammonds et al. 2013). The Dorsal embryo was stained using the 7A4 antibody (Whalen and Steward 1993) from the Developmental Studies Hybridoma Bank and imaged using a confocal microscope (see Methods).

Because prokaryotic promoters emerge relatively frequently, they present a unique system to explore how emergence differs in various types of sequences. For example, in three independent studies, we isolated nonpromoter DNA that was (i) randomly synthesized (Fuqua and Wagner 2026), (ii) from the *Escherichia coli* genome (Fuqua and Wagner 2026), (iii) from AT-rich regions of the *E. coli* genome called “Promoter Islands” (Fuqua et al. 2024), or (iv) from the ends of a family of pervasive transposons called IS3s (Fuqua and Wagner 2025). We created random mutagenesis libraries from these different sequences and screened their respective mutants for novel promoter activity using Sort-Seq (Peterman and Levine 2016) (Fig. 2a).

For each mutagenized sequence, we calculated the probability *P*_new_ that any given (random) mutation will generate de novo promoter activity. These values significantly differ between the categories of DNA (Fig. 2b, Kruskal–Wallis test, H = 88, *P* = 5.98 × 10^−19^). For example, we find that random DNA is ∼3 times more likely to acquire new promoter activity compared to genomic DNA (Fuqua and Wagner 2026) (Fig. 2b). The random and genomic sequences both lack hexamers resembling promoter motifs (ie −10 boxes, hence their lack of promoter activity), but differ in length (random, 150 bp; genomic, ∼127 bp) and AT content (random, 55.6 ± 6%; genomic, 46.7 ± 5%). However, after accounting for these variables, the *P*_new_ difference still holds (see Fig. S5 from Fuqua and Wagner (2026)). Thus, relative to randomly synthesized DNA, we argue that the *E. coli* genome has an emergence bias “against” creating new promoters or, conversely, that random DNA has an emergence bias “toward” creating new promoters, relative to the *E. coli* genome.

We also compared promoter emergence within the sequence categories. For example, the promoter island *P*_new_ values range from as low as 0.2% to as high as 41% (Fuqua et al. 2024) (Fig. 2b). For the mutagenized pieces of mobile DNA without promoter activity, these probabilities also range extensively (*P*_new_ = 2% to 23%) (Fuqua and Wagner 2025) (Fig. 2b). These studies demonstrate that relative to each other, groups of similar sequences can also vary in their propensity to give rise to promoters (Fuqua et al. 2024; Fuqua and Wagner 2025, 2026). We propose that these observations can also be described as “emergence biases” relative to their categories (Fig. 2c).

#### Eukaryotic Enhancer Emergence Biases

Enhancers are eukaryotic DNA sequences which control the spatial–temporal patterning of gene expression (Banerji et al. 1983) and encode binding sites for proteins (transcription factors, TFs) to bind and activate or repress transcription (Long et al. 2016). Multiple studies demonstrate how de novo enhancers can arise following point mutations (Eichenlaub and Ettwiller 2011; Rebeiz et al. 2011; Li et al. 2023), allowing us to also observe emergence biases of enhancers.

Similar to bacterial promoters, DNA sequences without detectable enhancer activity also appear to bias enhancer emergence. For example, Taskiran et al. recently used a sequence-to-function model called “DeepFlyBrain” (Janssens et al. 2022) to predict enhancer activity in *Drosophila* Kenyon cells (KCs) from given DNA sequences (Taskiran et al. 2024). The authors then created stepwise permutations in silico to 500 bp (enhancers are ∼100 to 300 bp in length (Struhl 2025)), nonenhancer sequences that incrementally improved their predicted scores (ie greedy walk). The model predicted that some sequences required just six point mutations to become “from scratch” KC enhancers (∼1.2% change), while others required up to 15 point mutations (∼3% change) and still had weak predicted strengths (Taskiran et al. 2024). While there are still limitations to predicting functions from point mutations using sequence-to-function models (Buel and Walters 2022; Pak et al. 2023), these simulations suggest that relative to each other, the former sequences have an emergence bias “toward” becoming enhancers, and the latter “against” becoming enhancers.

Relatedly, another recent study trained a sequence-to-function model based on ATAC-seq data to understand how chromatin accessibility has evolved within three species of *Drosophila* (Khodursky et al. 2023). The authors found that sequences exhibiting weak properties of open chromatin were more likely to become open in sister species. The authors called such regions “ancestrally poised” for acquiring novel enhancer activity. This poising is an example of DNA sequences exhibiting an emergence bias “toward” becoming enhancers.

Here, to further explore the emergence biases of enhancers, we used a similar sequence-to-function model called “DeepSTARR,” which predicts enhancer activity in *Drosophila* Schneider 2 (S2) cells (de Almeida et al. 2022). Using DeepSTARR, we first identified 228 nonenhancer sequences from randomly generated DNA, and 469 nonenhancer sequences from the *Drosophila melanogaster* genome. For each of these starting sequences, we quantified the likelihood of random mutants acquiring new enhancer activity (Fig. 1d) (one to ten point mutations per mutant; 2,000 mutants per starting sequence, see Methods). We find differences in emergence likelihoods within the random (*P*_new_ = 0.15% to 7.4%) and genomic (*P*_new_ = 0.0% to 6.9%) starting sequences, as well as between the sequence groups, with enhancers emerging nearly twice as frequently from mutants in random DNA versus genomic DNA (median *P*_new_ = 1.9% vs. 1.0%, Mann–Whitney *U* test, U = 74,036, *P* = 1.60 × 10^−16^) (Fig. 1e). These simulations suggest once again that there are biases underlying enhancer emergence.

In another study, we synthesized random libraries of DNA and screened them for enhancer activity in *Drosophila* embryos (Galupa et al. 2023). We found that the majority of sequences had detectable expression in the embryos, but their expression patterns were biased based on which TF binding sites the random DNA serendipitously encoded. This finding resonates with a recent study, which demonstrates that in *Drosophila*, de novo genes (ie those originating from noncoding DNA) are more likely to be regulated by a subset of specific TFs (eg Vis, Achi, and Jri) and, by extension, expressed in specific cell and tissue types (eg testis and accessory glands) (Peng et al. 2025).

These findings also reflect related studies that transposable elements (TEs) are biased toward recruiting specific TFs and can thus bias the emergence of tissue- or response-specific gene expression (Fig. 1f) (Lynch et al. 2015; Villar et al. 2015; Rech et al. 2019; Villanueva-Cañas et al. 2019; Ito et al. 2020). For example, three pervasive *Drosophila* TE families—*1360*, *Cr1a*, and *roo*—are each significantly enriched with binding sites for Tango (Rech et al. 2019; Villanueva-Cañas et al. 2019), which plays an important role in hypoxia response (Lavista-Llanos et al. 2002). This finding suggests that *Drosophila* TEs may have an emergence bias toward creating hypoxia-response elements. The *roo* elements are also enriched with binding sites for Dorsal—an early developmental TF—and a specific long interspersed element called Jockey, are enriched with binding sites for Caudal, another developmental TF (Villanueva-Cañas et al. 2019). Because these TFs are differentially expressed throughout development (Fig. 1f), we speculate that TE-based enhancers may be biased toward emerging within tissues that express these TFs.

Overall, the aforementioned studies demonstrate a proclivity of individual sequences, or groups thereof, to become enhancers relative to others. They additionally demonstrate a biased recruitment of specific TFs in de novo enhancers, controlling the tissues and cell types in which de novo enhancers are active. We propose describing this proclivity for different DNA sequences to become enhancers, and drive expression in particular cell types, as emergence bias.

#### Protein Emergence Biases

Novel protein-coding genes can evolve from previously noncoding sequences through a process known as de novo gene birth (Zhao et al. 2024). Once considered wholly implausible, the existence of de novo genes has now been demonstrated in organisms across the evolutionary tree, from plants (Zhang et al. 2019) to fungi (Vakirlis et al. 2018), humans (Knowles and McLysaght 2009), and bacteria (Fellner et al. 2015). A growing body of evidence suggests that pervasively translated small open reading frames (sORFs)—which naturally occur in different types of transcripts—provide the substrate for de novo gene birth (Ruiz-Orera et al. 2018) and serve as the foundation from which a proto-gene can evolve (Carvunis et al. 2012).

Such evolutionarily nascent, translated sORFs could have an emergence bias by being “preadapted,” that is, already exposed to selective pressures. Studies have predicted that as lowly expressed polypeptides are exposed to selection, deleterious ones (eg those prone to aggregate (Foy et al. 2019)) should be purged from the genome as soon as they appear (Masel 2006). Hence, the pool of expressed polypeptides would be enriched for those with a higher chance of evolving into a functional protein because they will not be toxic and, in turn, result in evolutionarily young genes having exaggerated gene-like properties. Consistently, low expression is an almost universal feature of young genes (Van Oss and Carvunis 2019), and young mouse and *Drosophila* proteins have more intrinsic structural disorder (ISD) than both older ones and random intergenic ORF-encoded polypeptides (Wilson et al. 2017; Heames et al. 2020). However, such trends are not found in all species (eg the opposite ISD trend is found in yeasts (Basile et al. 2017; Vakirlis et al. 2018), studies have reported no trends over time (Peng and Zhao 2024)), and this difference in trends is partly explained by genomic GC content (Basile et al. 2017).

Emergence biases may also prevent the aberrant expression of potentially damaging polypeptides. A recently identified mechanism relies on the presence of a C-terminal hydrophobic tail, which targets polypeptides for proteasomal degradation through a pathway that is also used for membrane targeting (Kesner et al. 2023). This property is abundant in polypeptides encoded by sORFs outside of known protein-coding regions but depleted within them. Another mechanism relies on the presence of degrons, short terminal motifs that target proteins for proteasomal degradation, and they also appear to be more prevalent within polypeptides encoded by noncanonical sORFs than canonical protein-coding genes or randomized controls (Casola et al. 2026).

**Fig. 3.**
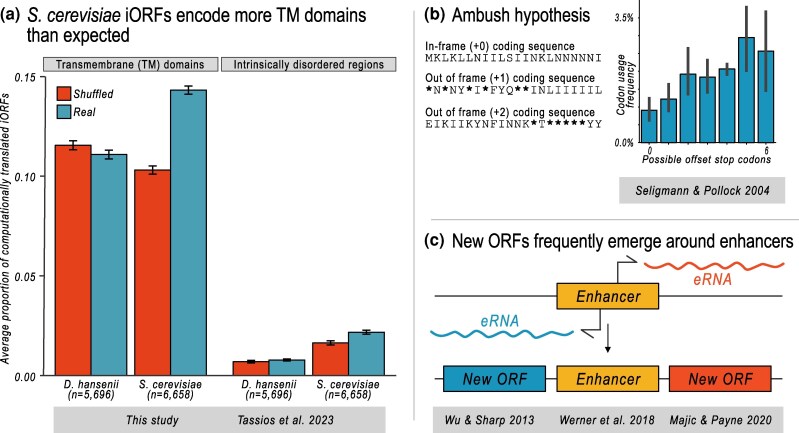
Emergence biases at the protein level. a) Average proportions of iORFs, full intergenic regions computationally translated into proteins after the removal of in-frame stop codons, and their shuffled counterparts (see Methods and Tassios et al. (2023)), in two species of budding yeasts, that constitute TM domains as predicted by Phobius and intrinsically disordered regions as predicted by IUPRED3. b) Left: the ambush hypothesis states that codon usage favors stop codons (shown with asterisks *) to conserve resources from ribosomal frameshifting. Top, an example yeast coding sequence; middle, the same sequence frameshifted +1 base; bottom, the same sequence frameshifted +2 bases. Right: the ambush hypothesis is supported by the positive correlation between codon usage versus the number of possible offset stop codons (see Methods and Seligmann and Pollock (2004)). c) Top: many mammalian enhancer sequences (center yellow rectangle) express bidirectional transcripts called eRNAs. Bottom: eRNAs appear to be a substrate for producing new ORFs.

The sequence of an sORF may bias the functions it can acquire. We previously showed that intergenic regions of the *Saccharomyces cerevisiae* genome have a surprisingly high potential to, if theoretically translated, encode polypeptides with computationally predicted protein transmembrane (TM) domains (Vakirlis et al. 2020). This bias also applies to the majority of *Saccharomycotina* yeasts (Tassios et al. 2023) and to a lesser extent to *Enterobacteriaceae* bacteria (Fesenko et al. 2025). In Fig. 3a (left), we show an example comparison of *S. cerevisiae*, which exhibits the bias, to *Debaryomyces hansenii*, a species that does not. Our most recent work (Vakirlis and Fuqua 2025) revealed that this bias is caused by the existence of polyA/T tracts, which are repetitive strings of adenine (AAAA…AAAA), thymine (TTTT…TTTT), and alternating adenine and thymine (TATA..TATA) within the sORF DNA sequence. These polyA/T tracts are abundant and enriched in intergenic regions and are known to function as nucleosome-depleted regions to enable transcription of surrounding genes. At the same time, it is possible that these polyA/T tracts are part of an unknown translation mitigation mechanism (Houghton et al. 2025) like the one recognizing hydrophobic CTDs (Kesner et al. 2023). Regardless, the presence of polyA/T tracts likely biases de novo proteins to be initially localized within specific intracellular locations and encode specific biochemical properties.

Consistently, a previous study has shown that *D. hansenii* has much shorter polyA/T tracts, which do not appear to be able to repulse nucleosomes (Tsankov et al. 2010), possibly an adaptation to the high salt concentration environments that this species inhabits. In contrast to protein TM domains that are predicted to be prevalent in intergenic regions of both species if translated, intrinsically disordered protein segments are predicted to be much rarer in both genomes and hence, while a weak enrichment can be seen in *S. cerevisiae* relative to shuffled controls, it is essentially inconsequential (Fig. 3a, right).

Protein sequences themselves also appear to exhibit emergence biases *against* creating novel transcripts. The ambush hypothesis is that selection favors out-of-frame stop codons within coding sequences through codon usage, effectively prematurely terminating frameshift-translated proteins. Such selection would conserve resources and energy from producing nonsense proteins and avoids nonsense proteins with cytotoxic functions (Seligmann and Pollock 2004). Indeed, we can computationally frameshift a yeast coding sequence and find many out-of-frame stop codons (Fig. 3b). While there are some criticisms of the model (Abrahams and Hurst 2018), one of the strongest arguments for the hypothesis is that there is a positive correlation between the frequency that a codon is used in the yeast genome versus the number of stop codons that the given codon could create if frameshifted (Seligmann and Pollock 2004). Here, we repeated the analysis in the *S. cerevisiae* genome and confirm the previously reported trend (Fig. 3b). Ultimately the extra out-of-frame stop codons create an emergence bias against the emergence of proteins with cytotoxic functions, as well as the use of frameshifted proteins as a source for evolutionary innovation.

#### Regulatory DNA as a Substrate for Emergence

A number of studies and models also support the idea that many de novo genes emerge from within or surrounding regulatory DNA (ie enhancers and promoters) (Wu and Sharp 2013; Carelli et al. 2018; Werner et al. 2018; Lee et al. 2024). For example, in addition to their role of interacting with promoters to transcribe genes, enhancers also produce bidirectional transcripts called enhancer RNAs (eRNAs) (Andersson et al. 2014) (Fig. 3c). These eRNAs can act as substrates for producing de novo genes (Wu and Sharp 2013; Werner et al. 2018), following a “transcription-first” model of gene emergence (Wilson and Masel 2011). For example, ∼47% of all mouse-specific intergenic ORFs (ie de novo mouse genes) are located within 500 bp of an enhancer and are more stably expressed than those not near or within an enhancer sequence (Majic and Payne 2020). Another study identified a set of mammalian enhancers that have undergone “repurposing” into promoter sequences to drive species-specific transcripts (Carelli et al. 2018). Collectively, these studies suggest that enhancers may have an “emergence bias” toward producing de novo genes.

Another model of de novo gene birth and the role of regulatory DNA is the cultivator model (Lee et al. 2024). Here, de novo genes emerge when mutations to the promoter sequence of a “cultivator” gene simultaneously increase fitness while creating a new transcript, such as an antisense long noncoding RNA (lncRNA). Because selection acts upon the expression of the cultivator gene, the frequency of this lncRNA can increase within a population. During or after fixation, the lncRNA has the opportunity to accumulate new mutations, which give it additional functions for selection to act upon. Similar to the enhancer model of gene birth (Fig. 3c), the cultivator gene model suggests that new ORFs emerge around or within preexisting regulatory DNA.

### Explanations for Emergence Biases

**Fig. 4.**
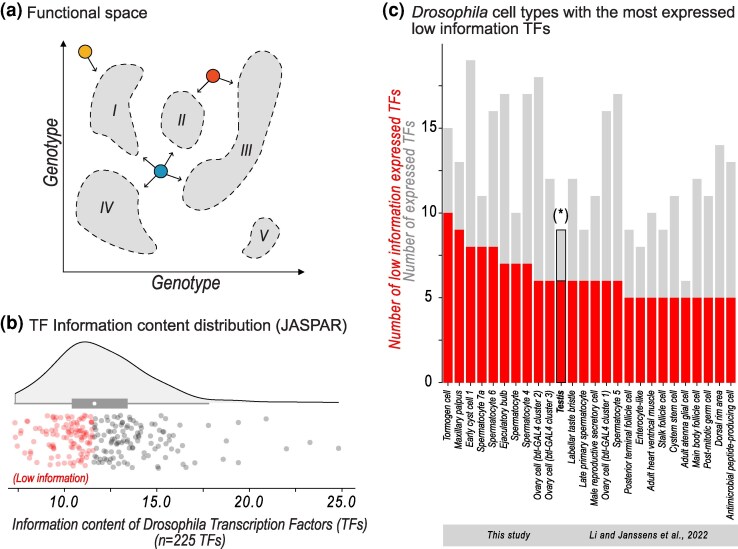
Information theory and sequence space influence emergence. a) A hypothetical representation of different functions (dashed gray shapes labeled I–V) in genotypic space (*x* and *y* axes). The colored circles correspond to three different DNA sequences in the genotypic space. The arrows show which functions these sequences are biased toward based on their proximity in the space. b) The information content of 225 TFs in *D. melanogaster* from the JASPAR database (Castro-Mondragon et al. 2022). TFs below the median are classified as “low-information” TFs and are labeled in red. See Data S1 for the identity of each TF and its respective information content. c) Bar plot of different *Drosophila* cell types from the Fly Cell Atlas (Li et al. 2022) and the number TFs (from panel b) expressed in each. See Data S2 for the remaining cell types.

Many cases of emergence biases can be explained by the topology and density of the genotype–function space. To illustrate this, we represent all possible DNA sequences within a high-dimensional genotype space, which we illustrate in Fig. 4a using two dimensions (for simplicity). Here, the closer two sequences are to each other, the more similar they are. Within this genotype space exist different clusters corresponding to unique biological functions. Depending on the given DNA sequence, some sequences will lie closer to a subset of functions versus others. For example (see Fig. 4a), the yellow genotype lies closest to the hypothetical Function I versus Functions II–V. In this example, point mutations in the yellow genotype will explore the surrounding genotype space, and a subset of these point mutants will overlap with (and thus acquire) Function I. In this case, the yellow genotype has an emergence bias toward Function I.

The genotype–phenotype map has been integral in exploring the relationship between RNA and its predicted 2D secondary structures and, by extension, RNA's evolution and emergence (Schuster et al. 1994; Fontana 2002; Cowperthwaite et al. 2008; Schaper and Louis 2014; Dingle et al. 2015; Dingle et al. 2022). Recent works have also experimentally demonstrated how these maps can influence evolvability (Papkou et al. 2023; Westmann et al. 2024; Herrera-Álvarez et al. 2025). For many functions, however, this genotype–phenotype map is (and will likely remain) largely hypothetical. Nonetheless, it can be useful to understand why some sequences have an emergence bias toward or against acquiring particular functions. For instance, DNA sequences are more likely to become promoters if they contain motifs or signatures that moderately resemble canonical promoters (Fuqua and Wagner 2025, 2026). They are also more likely to encode promoters as their AT content increases (Warman et al. 2020; Fuqua and Wagner 2026). Because of this resemblance, such sequences would lie close to the boundary of the “promoter function” space in the map, just like the yellow genotype is to Function I (see Fig. 4a). As another example, transposon coding sequences can coincidentally encode binding sites for particular TFs (Rech et al. 2019; Villanueva-Cañas et al. 2019) (see Fig. 2f). Encoding such sites decreases the distance between the transposon and the boundaries of the “enhancer” or “promoter” functional space.

The bias toward encoding TM domains within yeast intergenic regions can also be explained using the genotype–phenotype map. This is because highly repetitive polyA/T DNA tracts, and the respective amino acids they encode (Phe, Lys, Tyr), enable the formation of TM helices largely due to their hydrophobicity (Prilusky and Bibi 2009; Vakirlis et al. 2020) and not their specific amino acid sequences. Because the intergenic regions of the yeast genome are enriched with repetitive genetic sequences of A's and T's (Vakirlis and Fuqua 2025)—whose primary function might be to hinder nucleosome binding (Segal and Widom 2009)—intergenic regions would also lie close to the TM domain function space. Note: these polyA/T DNA tracts are removed relatively quickly during the evolutionary time following the initial emergence of a gene and are extremely rare in conserved genes, including those encoding TM domains (Vakirlis and Fuqua 2025). Thus, the low-information content of these polyA/T DNA tracts is only relevant to their role in the emergence of novel proteins and is not a feature of TM proteins more generally.

The influence of AT content on emergence is not just limited to TM domains. A recent study explored the role of AT content in de novo gene birth across over 3,000 genomes (Roginski et al. 2026). The scientists concluded that de novo proteins have an emergence bias toward emerging from GC-rich regions, as protein folding potential increases with GC content through the recruitment of amino acids encoded by GC-rich codons (Roginski et al. 2026).

Furthermore, some functions can be, by their very nature, more easily accessible by different sequences. For example, promoters and enhancers operate through protein binding sites for RNA polymerases and TFs. The “information content” of these binding sites differs for each factor, where some TFs bind to very specific sequences (high information), while others to a variety of degenerate sequences (low information) (Schneider et al. 1986; Wunderlich and Mirny 2009; Stewart et al. 2012). In other words, protein binding “specificity” influences which TFs regulate a given DNA sequence (Wunderlich and Mirny 2009). In truly random DNA, this information content is directly proportional to the probability that a TF will bind a given sequence (Wunderlich and Mirny 2009; Galupa et al. 2023; Fuqua and Wagner 2026). The differences in the information content of TF binding may also explain why de novo enhancers and promoters use particular subsets of (low-information) TFs (Galupa et al. 2023; Fuqua and Wagner 2026) and why de novo genes recruit less TFs compared to older genes (Witt et al. 2021; Peng et al. 2025). Low-specificity binding sites may also enhance the evolvability of enhancers (Kurafeiski et al. 2019), as these sites increase the number of neutral mutations new enhancers can accumulate, allowing the sequence “to explore” additional, novel regulatory interactions (Wagner 2008, 2012; Kurafeiski et al. 2019).

Here, to illustrate just how much TF binding specificity could influence enhancer emergence, we acquired a set of 225 TF binding motifs for *D. melanogaster* (Castro-Mondragon et al. 2022) and calculated their respective information contents (Fig. 4b), which range from 7.3 to 24.8 bits (median 11.6 bits), similar to previous reports for other eukaryotic TF binding motifs (Wunderlich and Mirny 2009). We classified TFs with less than 11.6 bits (50th percentile) as “low-information” TFs, which will bind more frequently to DNA sequences. These low-information TFs are thus more likely to be recruited for de novo enhancer emergence. See Data S1 for the TFs and their respective information contents.

Because TFs are also differentially expressed, we can extrapolate from the information content to estimate in which cell types, information theory predicts new enhancers are more likely to emerge from. To this end, we used the Fly Cell Atlas, which contains single-cell RNA-seq expression data for 246 cell types in *D. melanogaster* (Li et al. 2022). We used TF transcript levels to infer whether or not each of the 225 TFs is expressed in each cell type, and then counted how many of the low-information TFs are expressed in each cell type (see Methods). In Fig. 4c, we plot the cell types that express at least five low-information TFs (see Data S2 for the complete set of counts per cell type). Thus, based on information theory and these data, we hypothesize that new enhancers may be biased toward producing expression specifically in these cell types compared to other cell types. They include germline cells (eg spermatocytes and oocytes), somatic support cells, and various epithelial and secretory cell types (Note: the logic behind our hypothesis may be an oversimplification, as it ignores the concentration of the TFs in the nucleus, as well as the presence or absence of competitive and synergistic TFs). Interestingly, the testes are also part of this subset, which could be linked to the observation that many de novo genes are expressed in the *Drosophila* testes (Peng et al. 2025).

### Future Directions and Conclusions

Here, we provide evidence for emergence biases in the context of de novo promoters, enhancers, and proteins. Such biases dictate the likelihood of different sequences acquiring these functions relative to each other or relative to random DNA. They appear to be governed by the information content encoded within these functions, and the space a sequence occupies. We believe that a critical question for future investigations is whether such biases are more commonly the result of selective filtering or mutational biases, either for some specific functionality or because they make genomes more evolvable, which can be beneficial over an unbiased “naive” state. We see no reason why emergence biases would be limited to promoters, enhancers, and TM domain functions, nor do we assume that there are no other mechanisms underlying them.

Selection can only act upon existing genetic variation. Like all biases in molecular biology, skewing or limiting what standing variation can or cannot exist, by extension, biases evolution itself. We can envision how emergence biases can strongly influence which phenotypes mutations can produce, potentially canalizing evolution in particular directions. However, we do not know the extent to which emergence biases can impact evolution at this time. We encourage others to think about these biases and to build upon the concept.

## Methods

### DeepSTARR Enhancer Predictions for *Drosophila* S2 Cells

To calculate enhancer *P*_new_ values for the different categories of DNA, we first isolated nonenhancer “parent” sequences. For the random DNA parents, we randomly generated a library of 5,000 DNA sequences, each 249 bp in length in silico. From each of these candidate sequences, we used the following DeepSTARR command to predict the developmental enhancer activity of the 5,000 sequences:

python DeepSTARR_pred_new_sequence.py -s query_sequences.fasta -m DeepSTARR.model

From the predictions, we selected 228 parents from the randomly generated DNA sequences with no predicted developmental enhancer activity, by selecting sequences ranging with prediction scores from −0.1 to +0.1 RNA log2 fold-change (FC) levels. For each parent, we then computationally generated 2,000 unique mutants with one to ten random point mutations each. We then predicted the developmental enhancer activity for each of these mutant “daughter” sequences using the previous DeepSTARR command and calculated the parent's respective *P*_new_ value by dividing the number of daughters with predicted enhancer activity (log2 FC values > +1.0) by the total number of daughters (2,000 daughters each).

To isolate genomic sequences without enhancer activity, we randomly selected 5,000 genomic sequences from the dm6 assembly (dos Santos et al. 2015), sampling evenly and exclusively within chromosomes 2L, 2R, 3L, 3R, X, and Y. We identified 469 genomic sequences with log2 FC levels between −0.1 and 0.1 using the previous DeepSTARR command and subsequently analyzed respective mutagenesis libraries as described above.

### Identifying Cell Types Expressing Low-Information Content TFs

We acquired a set of 290 *Drosophila* TF binding matrices from the JASPAR database (Castro-Mondragon et al. 2022) (core insecta, nonredundant PFMs) using the following link: https://jaspar.elixir.no/downloads/. If multiple matrices existed for a given TF, we chose the matrix with the highest information content. We acquired the TF expression data for *Drosophila* cell types from Table S3 of the Fly Cell Atlas (Li et al. 2022). The data contained 246 unique cell types with 501 unique TFs.

We filtered the Fly Cell Atlas dataset to only focus on TFs present in both datasets (*n* = 225 TFs). See Data S1 for the information content values (in bits) for each of the 225 TFs. To define a TF as “expressed” within each cell type, we noted if it appeared within any of the three columns of the supplemental Table S3 from Fly Cell Atlas: “high_genes (expr ratio > 50%),” “medium_genes (50% > expr ratio > 5%),” or “low_genes (expr ratio < 5%)”. With this approach, a TF is either present or absent in each cell type, and we did not consider transcript dosage.

For each cell type, we then counted how many of the 225 shared TFs were present in each cell type (see Fig. 4c, gray bars). We then isolated a subset of the TFs below the 50th percentile of all information content values, which we call the “low-information TFs” and repeated the counting procedure for each cell type (see Fig. 4c, red bars). Figure 4c only displays a subset of the data. See Data S2 for the data on all cell types.

### Dorsal Antibody Staining

We added a strain of w^1118^  *D. melanogaster* lines to an egg collection chamber and collected embryos after an overnight incubation. We washed the collected embryos with saline, and dechorionated them using a 50% bleach solution for 90 s, followed by washing with water. We then transferred the embryos to scintillation vials and fixed the embryos with 700 μL 16% PFA, 1.7 mL PBS/EGTA, and 3.0 mL 100% heptane for 25 min while shaking at 250 rpm. After fixation, we removed the lower phase of the solution and added 100% methanol to carry out an isotonic shock, followed by subsequent vortexing. We removed embryos from the interphase and upper phases of the vial.

We stained the embryos using an antidorsal antibody (7A4, DSHB) and conjugated the antibody with a secondary Alexa Fluor antibody (1:500, Invitrogen). The antidorsal 7A4 antibody (Whalen and Steward 1993) was deposited to the DSHB by Steward, R. (DSHB Hybridoma Product antidorsal 7A4). We mounted the fixed and stained embryos using Prolong Gold (Thermo Fischer Scientific) with DAPI and imaged the embryos using a Zeiss 880 confocal microscope (Zeiss, Germany).

### Protein-Level Biases in iORFs

Proportions of amino acids predicted to be in TM protein domains in real and single-nucleotide shuffled iORFs in *S. cerevisiae* and *D. hansenii* were obtained from Tassios et al. (Tassios et al. 2023). The same iORF sequences were given as input to IUPRED3 (Erdős, Pajkos, and Dosztányi 2021) with the *-long* parameter to obtain the numbers of amino acids predicted to belong in intrinsically disordered regions (with probability above 0.5). For each sequence, this number was divided by the sequence length to obtain the proportion.

### Ambush Hypothesis

We calculated codon frequencies from all annotated ORFs from the *S. cerevisiae* genome (R64 assembly, NCBI: GCF_000146045.2). To calculate the number of possible offset stop codons, we followed the procedure in the original publication (Seligmann and Pollock 2004). Briefly, for each of the 64 possible codons, we computationally generated a set of all DNA sequences with two variable nucleotides upstream and downstream of each codon (NN + codon + NN, where N = A, C, G, or T). Within each of these 7 bp sequences, we counted how many unique stop codons (standard genetic code: TAA, TAG, and TGA) occur within positions 1:3, 2:4, 4:6, and 5:7.

## Supplementary Material

evag195_Supplementary_Data

## Data Availability

We provide the data and scripts used to create the figures and analyses in this study at the GitHub repository: https://github.com/tfuqua95/emergence_bias.
